# Effects of Early Enteral Glutamine Supplementation on Intestinal Permeability in Critically Ill Patients

**DOI:** 10.5005/jp-journals-10071-23218

**Published:** 2019-08

**Authors:** Zahra Vahdat Shariatpanahi, Ghazaleh Eslamian, Seyed Hossein Ardehali, Ahmad-Reza Baghestani

**Affiliations:** 1 Department of Clinical Nutrition and Dietetics, Faculty of Nutrition and Food Technology, National Nutrition and Food Technology Research Institute, Shahid Beheshti University of Medical Sciences, Tehran, Iran; 2 Student Research Committee, Department of Clinical Nutrition and Dietetics, Faculty of Nutrition and Food Technology, National Nutrition and Food Technology Research Institute, Shahid Beheshti University of Medical Sciences, Tehran, Iran; 3 Department of Anesthesiology and Critical Care, Shohadaye Tajrish Hospital, Shahid Beheshti University of Medical Sciences, Tehran, Iran; 4 Department of Biostatistics, Physiotherapy Research Center, Faculty of Paramedical Sciences, Shahid Beheshti University of Medical Sciences, Tehran, Iran

**Keywords:** Antiendotoxin immunoglobulin, Endotoxin, Enteral nutrition, Glutamine, Zonulin

## Abstract

**Background and aims:**

Enteral administration of glutamine has been proposed as an effective recovery of intestinal barrier function. This amino acid has a modulating effect on the reducing bacterial translocation, which can influence immune functions of the intestine. The objective was to evaluate the effects of early enteral glutamine supplementation on intestinal permeability in critically ill patients.

**Materials and methods:**

A total of 80 critically ill patients older than 18 years were randomly assigned to one of two groups according to the stratified blocked randomization by age and admission category. Consecutive participants took enteral formula plus 0.3 g/kg/day glutamine powder or enteral formula plus maltodextrin during the ICU stay for a maximum of 10 days. Plasma glutamine, endotoxin, zonulin, and antiendotoxin immunoglobulin (Ig)G/IgM concentrations were measured on days 5 and 10 of intervention.

**Results:**

Out of 80 participants, 36 patients in the glutamine group and 34 patients in the control group were included in the analysis of the outcomes. Enteral glutamine significantly reduced plasma zonulin concentration up to 40% during 10 days. This reduction was significantly greater compared with that of the placebo group (*p*<0.001). Endotoxin concentration decreased in both groups; this reduction was significantly greater in the glutamine group (*p* = 0.014). The antiendotoxin IgM and IgG antibody levels increased in the glutamine group but decreased in the control group (*p* <0.001). There were no significant differences in clinical outcomes between two groups.

**Conclusion:**

Early enteral glutamine supplementation led to a declined intestinal permeability in critically ill patients.

**How to cite this article:**

Shariatpanahi ZV, Eslamian G, Ardehali SH, Baghestani AR. Effects of Early Enteral Glutamine Supplementation on Intestinal Permeability in Critically Ill Patients. Indian J Crit Care Med 2019;23(8):356–362.

## INTRODUCTION

The altered gut function is a common problem in the critically ill patients.^1,2^ Delayed gastric emptying, abnormal motility patterns, and impaired intestinal barrier integrity are commonly reported in the intensive care unit (ICU).^1^ The leaky gut may play an important role in the development of catabolic states in critically ill patients.^3^ It has been proposed that bacterial translocation and gut-origin sepsis may be involved in the pathogenesis of systemic infectious complications and multiple organ deficiency syndromes.^4^ Conditions leading to intestinal hypoperfusion such as hemorrhagic shock, trauma, burns, pancreatitis, and major abdominal operations cause breakdown of the intestinal mucosal barrier.^3^

Previous studies have illustrated a link between glutamine supplementation and preservation of enteric mucosa integrity.^5^ Glutamine is the preferred metabolic fuel for enterocytes and plays a key role in maintaining intestinal barrier integrity and function.^6^ Although glutamine is classified as a nonessential amino acid, it may become conditionally indispensable in hypermetabolic states.^7^ Glutamine is reduced in critically ill patients.^8^ Experimental studies determined that depletion of glutamine can be viewed as a compromised body defense system, which leads to villus atrophy, decreased expression of tight junction proteins in several conditions, and increased intestinal permeability.^6^ Glutamine has a modulating effect on the reducing bacterial translocation, which can influence immune functions of the intestine.^8^ Intestinal permeability is most commonly measured by fractional urinary excretion of orally ingested probes, circulating biomarkers of gut barrier function, and circulating zonulin level, the only physiological modulator of intercellular tight junctions.^9^ Assessing the effects of enteral glutamine on intestinal permeability in critically ill and burned patients shows diverse results.^10–14^

Today, supplementation of glutamine is both a confusing and controversial topic. According to the last revision of American Society for Parenteral and Enteral Nutrition guideline, enteral glutamine exerts a trophic effect in maintaining gut integrity. However, supplemental enteral glutamine is not recommended to be added to an enteral nutrition (EN) regimen routinely in critically ill patients.^15^

We hypothesize that after enteral glutamine administration, plasma antiendotoxin immunoglobulin G (IgG) and IgM would increase while plasma zonulin and endotoxin levels decrease as the biomarkers of intestinal permeability. Therefore, to evaluate this hypothesis, a clinical trial was established to determine effects of early enteral glutamine supplementation on intestinal permeability in ICU-hospitalized patients.

## MATERIALS AND METHODS

### Study Design and Participants

The design of the current study was a randomized, double-blind, placebo-controlled trial. In this study, 80 critically ill patients were recruited between April and October 2017 from general ICU of a university hospital. After a full review of the inclusion and exclusion criteria and explanation of the risks and benefits of the study, critically ill patients meeting the inclusion criteria of the study were enrolled. The study was approved by the related ethics committee and performed in accordance with the ethical standards laid down in the 1964 Declaration of Helsinki and its later amendments. The funder of the current study had no role in its design or conduct; in the collection, management, analysis, or interpretation of the data; or in the preparation, review, or approval of the manuscript.

Consecutive adult patients (>18 years old) were screened for eligibility according to the following inclusion criteria: Start of study intervention within 48 h after ICU admission; having the EN for at least 72 h aiming for full EN and receiving at least 80% of enteral formula during the first 48 h; and submitting written informed consent by the patients or their legal representative. On the other hand, the patients were excluded for one or more of the following criteria: >24 h from admission to the ICU; requiring other specific EN for medical reason such as renal or liver failure; death or discharge before day 5; any absolute contraindication to receive EN; pregnancy or lactating; body mass index less than 18 kg/m^2^ or greater than 40 kg/m^2^; not being expected to be in ICU for more than 48 h due to imminent death; liver cirrhosis –Child's class C liver disease; seizure disorder requiring anticonvulsant; history of allergy or intolerance to the study product components; receiving glutamine within two weeks before starting the study; and enrollment or planned enrollment in a related ICU interventional study.

Subject participation in the study was prematurely discontinued in the following situations: further participation was a health risk for the patient; a subject or legal representative who gave informed consent decided to resign from further participation in the study; and a patient might require a hospital transfer.

### Randomization, Masking, and Interventions

**Fig. 1:**
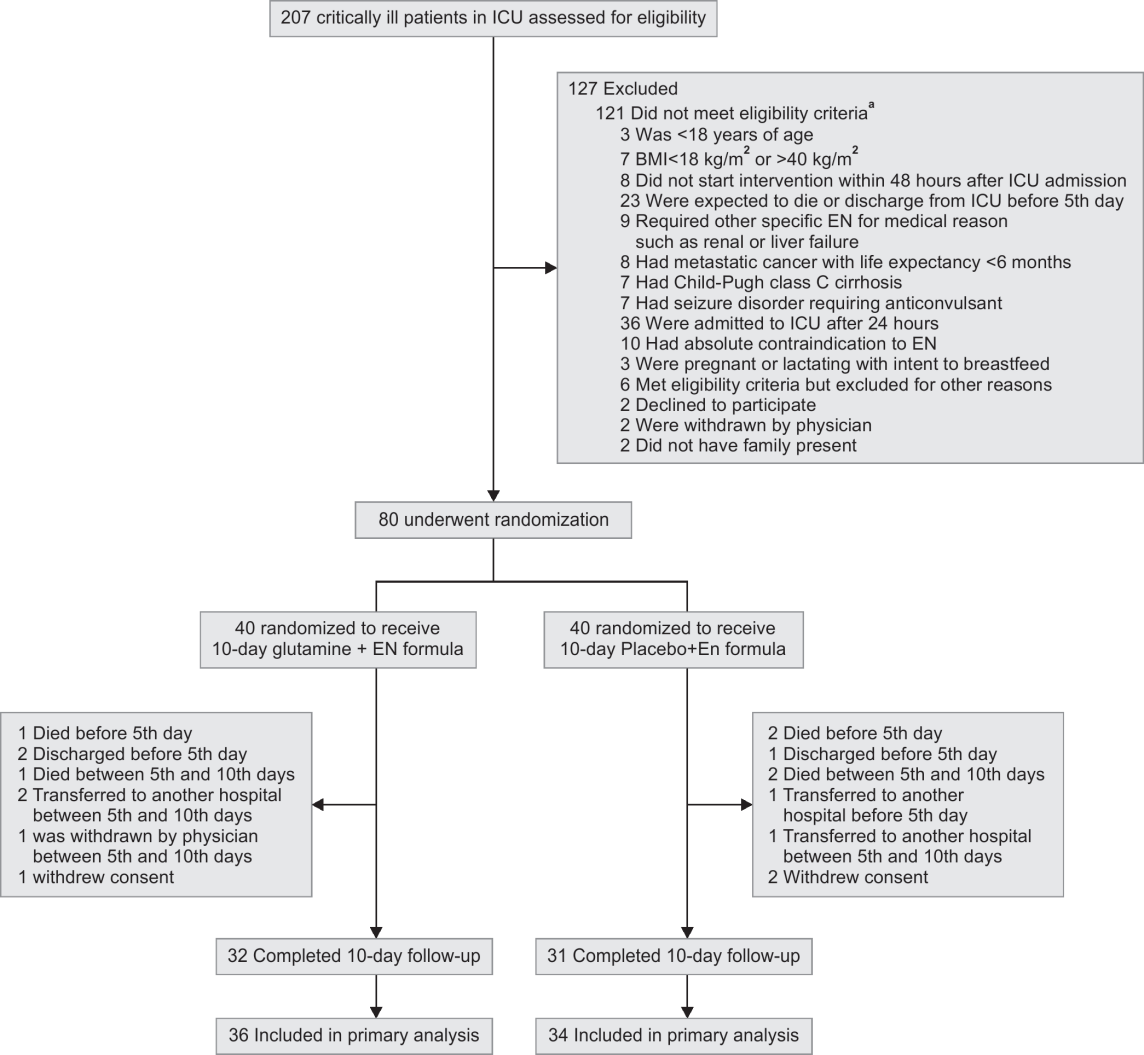
Study enrollment and randomization of critically ill patients

Figure 1 presents the details about the recruitment, randomization, and follow-up of the current study. Randomization was stratified by age (18–65 and ≥ 65years) and admission category (medical, surgical, and trauma). Patients that meet all the inclusion criteria and none of the exclusion criteria were assigned using computer-generated randomization to receive 10 days of enteral formula plus 0.3 g/kg/day glutamine powder or isocaloric enteral formula plus maltodextrin. ENTERA Meal formula (54.6% carbohydrate, 14% protein, and 31.6% fat), maltodextrin, and glutamine were provided by Karen Pharma & Food Supplement, Co., Tehran, Iran. The group assignments were concealed in sealed envelopes and opened at enrollment by somebody who was blinded to all baseline assessments.

### Administration of Intervention

Study product administration was started within 48 h after admission at the ICU and ended at discharge from the ICU or at end of the study (day 10). Planned enteral tube feeding (nasogastric or gastrostomy tube feeding) was administered after stabilizing the hemodynamic condition of each patient. Administration of formula was done by the intermittent method every four hours. The volume of administered tube feed was determined based on the calorie needs and tolerance of patients by starting from 50 mL. Through the first 24 h, if the patient could tolerate the feeding, the volume of administration was increased to the goal volume. The supplements were administrated three times a day. General recommended energy and protein intakes for critically ill patients –based on weight and metabolic condition– were 25–30 kcal/kg and 1.2–2 g/kg, respectively.^15^ According to the protein calculation, if a patient had required an extra amount of protein, modular protein supplements would have been added to the enteral formula. The volume and calorie intake were recorded on a daily basis. The volume ratio (VR) was calculated to determine the efficacy of daily nutritional administration: VR (%) = (volume received/volume prescribed) × 100.

## PROCEDURES

Baseline measurements were performed before starting EN with study product on day 1. At the screening visit, age, sex, length, medical history, preexisting conditions, medication use, enteral route, feeding regimen, date of hospital and ICU admission, and diagnosis for ICU admission were recorded. Medication and nutritional supplements used from two weeks before start study product until discharge from the ICU or the end of the study were also recorded. At baseline, the Acute Physiology and Chronic Health Evaluation (APACHE II),^16^ Sequential Organ Failure Assessment (SOFA) score,^17^ and Nutrition Risk score in the Critically Ill (NUTRIC)^18^ were determined over the first 24 h after admission at the ICU. From the start of feeding study product until day 10, the adverse events, gastrointestinal intolerance, the net amount of tube feeding intake, and mortality were assessed on a daily basis.

Venous blood samples were taken at baseline within 2 h before the start of intervention and between 7 am and 9 am on day 5 and Day 10 for laboratory data. Blood samples were collected in EDTA-containing tubes and plasma was separated by centrifugation at 3,000 rpm for 10min at 4°C. Plasma samples were snap-frozen and stored in small aliquots at –80°C to prevent repeated freezing and thawing until the determination of laboratory data.

### Primary and Secondary Outcomes

Plasma endotoxin, zonulin, and antiendotoxin IgG and IgM concentrations were the primary outcome measures. Endotoxin concentrations in plasma were measured by a commercially available quantitative chromogenic endpoint Limulus Amebocyte Lysate (LAL) QCL-1000 kit (Lonza, Walkersville, MD).^19^ Enzyme-linked immunosorbent assay (ELISA, Zonulin ELISA Kit, Immundiagnostik, Bensheim, Germany) was used to determine plasma zonulin concentration. Plasma antiendotoxin core antibody IgG and IgM concentrations were determined by an enzyme-linked immunosorbent assay (Chromogenix, Quadritech Diagnostics, Epsom, UK). All assays were performed according to manufacturers’ manual; tests were carried out in duplicates. The glutamine concentration in plasma was measured using the high-performance liquid chromatography, as described previously.^20^

Secondary outcomes included incidence of sepsis according to the American College of Chest Physicians and the Society of Critical Care Medicine,^21^ mortality at ICU, length of stay (LOS) in ICU, and incidence of gastrointestinal complications such as abdominal distention, vomiting, diarrhea, and constipation.

### Statistical Analysis

Data were analyzed by applying the SPSS software (version 20; Chicago, IL, USA). All hypothesis tests were two-tailed, with P<0.05 denoting the statistical significance. Data were presented as frequencies and percentages, means and 95% confidence intervals (CIs) or standard deviations, or medians and interquartile ranges (IQR), when appropriate. Chi-square or Fisher exact tests were used to evaluate the differences in the distribution of categorical variables, and Mann–Whitney test was used to check the differences in the distribution of continuous variables between intervention and control, as most of the variables were highly skewed and not normally distributed. The data were analyzed according to the intention-to-treat (ITT) principle. Participants missing laboratory measurements of the primary and secondary outcome measures were imputed using the regression method. Means and 95% CIs for changes from baseline in primary outcomes were compared at day 5 and day 10 using the ANCOVA.

## RESULTS

### Recruitment and Follow-up

Patient screening, enrollment, and retention by treatment groups are shown in Figure 1. Out of 80 participants, 32 patients in the glutamine group (80%) and 31 patients in the control group (77.8%) completed the 10-day follow-up of the study. Baseline characteristics of participants completing the study were similar to those who did not complete the protocol of the study. There were 70 patients in the final ITT analysis.

**Table 1:** Baseline characteristics of study patients

*Characteristic*	*Glutamine group n = 36*	*Control group n = 34*	*p value*
Age, yr, median (IQR)	62 (53–70)	64 (53–70)	0.728*
Sex, No. (%)			0.782**
Male	20 (55.6)	20 (58.8)	
Female	16 (44.4)	14 (41.2)	
Admission category, No (%)			0.967**
Medical	17 (47.2)	15 (44.1)	
Surgical	12 (33.3)	12 (35.3)	
Trauma	7 (19.5)	7 (20.6)	
Admision scores, median(IQR)			
APACHE-II	24 (19–47)	24 (19–37)	0.888*
NUTRIC	5 (4–6)	5 (4–6)	0.485*
SOFA	6 (5–7)	6 (5–7)	0.779*
Baseline blood levels, median(IQR)			
Albumin, g/dL	2.7 (1.6–3.3)	2.5 (1.7–4.4)	0.612*
Glutamine, mg/dL	373 (309–411)	387 (298–466)	0.879*

* Mann–Whitney test

**X^2^ test or Fisher exact test

APACHE-II, Acute Physiology and Chronic Health Evaluation; ICU, intensive care unit; IQR, interquartile range; NUTRIC, Nutrition Risk in the Critically Ill; SD, standard deviation; SOFA, Sequential Organ Failure Assessment;

**Table 2:** Feeding details of study patients

*Enteral feeding administration*	*Glutamine n = 36*	*Control n = 34*	*p value*
Time from ICU admission to study EN start, median (IQR), hr	28 (18–41)	30 (19–41)	0.510*
Duration of administration, median (IQR), d	10 (8–10)	10 (7–10)	0. 808*
Mean planned energy intake, median (IQR), kcal/d	1497 (1447–1584)	1506 (1408–1594)	0.809*
Mean planned protein intake, median (IQR), g/d	73 (65–83)	72 (66–80)	0.426*
Mean energy intake, median (IQR), kcal/d	1375 (1218–1483)	1352 (1240–1486)	0.261*
Mean protein intake, median (IQR), g/d	64 (57–80)	61 (56–71)	0.101*
VR at day 1, median (IQR), %	86 (83–92)	85 (83–90)	0.545*
VR at day 5, median (IQR), %	89 (86–94)	88 (85–91)	0.219*
VR at day 10, median (IQR), %	90 (83–96)	86 (82–94)	0.173*
Patients with supplemental parenteral nutrition, No (%)	5 (13%)	4 (11 %)	0.537**

* Mann–Whitney test

**X^2^ test

EN, enteral nutrition; ICU, intensive care unit; IQR, interquartile range; VR, volume ratio

### Baseline Characteristics of the Patients

Baseline demographic and medical characteristics are presented in Table 1. The study participants had a mean age of 59 years and 57% of them were male. As a result of the stratified randomization design, the age and admission category distribution were similar in the both groups.

The nutritional data of patients in both groups during the intervention are shown in Table 2. No statistically significant differences were observed between study groups in mean duration of study product administration, the time of starting nutritional support, the energy intake, the protein intake, or total volume of study product administered.

The baseline median (IQR) plasma glutamine of participants was 483 (418–516) and 494 (409–577) μmol/mL in the glutamine and control groups, respectively, demonstrating no significant difference (*p* = 0.503). At the end of the 10-day treatment period, median [IQR] plasma glutamine levels were significantly higher in the glutamine group compared with the control patients (588 μmol/mL [462–707 μmol/mL] versus 472 μmol/mL [440–536 μmol/mL], P=0.006). No adverse events were attributed to glutamine supplementation.

### Primary Outcome

The baseline plasma levels of intestinal permeability markers of the two groups were similar (*p*<0.05). Table 3 presents the mean changes of plasma levels of intestinal permeability markers. Plasma endotoxin concentration decreased in both groups on days 5 and 10; however, the reduction in the glutamine group was significantly greater than that in the placebo group. The change in the endotoxin concentration on days 5 and 10 showed a significant difference between those treated with glutamine or placebo. Furthermore, glutamine supplementation produced a significant decrease in the plasma zonulin concentration on days 5 and 10. Plasma levels of IgG and IgM antibodies increased in the glutamine group significantly on days 5 and 10 compared with the placebo group. However, at the end of the 10-day treatment period, a significant decrease in IgG and IgM antibodies levels was seen in the control group.

**Table 3:** Mean changes (95% CIs) from baseline in intestinal permeability outcomes by treatment group

*Change from baseline*	*Glutamine n = 36*	*Control n = 34*	p value*
Plasma endotoxin concentration (EU/mL)			
Day 5	–0.26 (–0.36, –0.16)	–0.09 (–0.25, 0.07)	0.002
Day 10	–0.40 (–0.52, –0.28)	–0.24 (–4.69, –0.01)	0.014
Plasma zonulin concentration (ng/mL)			
Day 5	–1.13 (–1.59, –0.67)	0.39 (0.06, 0.71)	<0.001
Day 10	–2.06 (–2.78, –1.33)	0.85 (0.17, 1.53)	<0.001
Plasma antiendotoxin IgG (MU/mL)			
Day 5	22.58 (13.27, 31.90)	–7.21 (–16.29,1.88)	<0.001
Day 10	52.03 (32.39, 71.67)	–29.79 (–39.96, –19.63)	<0.001
Plasma antiendotoxin IgM (MU/mL)			
Day 5	4.14 (–0.98, 9.26)	–7.91 (–13.70, –2.13)	0.005
Day 10	15.44 (6.61, 24.28)	–16.71 (–25.45, –7.96)	<0.001

*Based on an ANCOVA model that regressed changes from baseline on treatment group, baseline value of the outcome, albumin and energy intake

**Table 4:** Comparison of gastrointestinal complication diagnosed after initiation of intervention between groups

*Gastrointestinal complication, No (%)*	*Glutamine n = 36*	*Control n = 34*	p value*
Abdominal distention	11 (30)	11 (32)	0.538
Vomiting	3 (8)	3 (9)	0.635
Diarrhea	3 (8)	9 (26)	0.044
Constipation	6 (17)	13 (38)	0.039

*X^2^ test, Fisher axact test

### Secondary Outcomes

The results obtained from the analysis of gastrointestinal complication are presented in Table 4. As can be noted, there was no significant difference in terms of the incidence of abdominal distention and vomiting between both groups. The incidence of diarrhea was significantly lower in the glutamine group (8%) than in the control group (26%). The incidence of constipation was also significantly lower in the glutamine group (17%) than in the control group (38%).

There were no statistically significant differences in the incidence of new severe sepsis between groups. Overall, 5.6% of those in the glutamine group (95% CI; 0–13.9%) versus 11.8% in the control group (95% CI, 2.8–23.5%; *p* = 0.309) had new severe sepsis. There were no statistically significant differences for mortality at ICU between the glutamine group (19%, 95% CI; 8-33%) and the control group (12%, 95%CI; 3–24%; *p* = 0.378). The median (IQR) LOS in ICU was 10 (8–20) days and 13 (7–29) days for the glutamine group and the control group, respectively (*p* = 0.646).

## DISCUSSION

The current study provided valuable information on whether the administration of enteral glutamine could alter intestinal permeability in critically ill patients. Assessing the plasma endotoxin, zonulin, and antiendotoxin IgG and IgM concentrations in this study showed that intestinal permeability was improved after a short period of glutamine-supplemented enteral feedings. In addition, glutamine supplementation was associated with a significant decrease in the incidence of diarrhea and constipation. No effect of glutamine was observed on any other gastrointestinal complications, mortality, and LOS in ICU. Both groups were comparable in terms of baseline characteristics. Most patients tolerated EN and achieved the planned caloric goal within the first 2 days of treatment.

Glutamine as a major fuel source for both enterocytes and colonocytes plays a critical role in activating the mammalian target of rapamycin cell signaling in enterocytes. Therefore, it is necessary for the maintenance of intestinal structure in both normal and stressed states.^22^ During critical illness, nutrient deficiencies can further be exacerbated probably due to the impaired immune function and a higher incidence of developing infectious complications, multiorgan failure, and death. Lower plasma and skeletal muscle glutamine levels contribute to immune dysfunction, indicating elevated glutamine needs in critically ill patients.^23^ Glutamine may augment immune system response by lowering the incidence of bacteria and toxin translocation from the intestinal lumen to the systemic circulation.^24^ The effects of glutamine on intestinal permeability and its role in maintaining functional integrity of the gut have been investigated in humans and animal models.^25^ In most previous clinical trials in critical care, urinary lactulose and mannitol ratio (L/M) was determined as a gut permeability marker.^10,12–14^ Dual sugar absorption tests have some technical limitations. These limitations are related to timing of specimen acquisition, handling of urine, and assay performance.^26^ Some studies have evaluated the plasma endotoxin level as a marker of intestinal permeability with enteral glutamine supplementation in severe burn patients.^11,12^ In the present study, we were able to demonstrate the significant positive effects of enteral glutamine supplementation on intestinal permeability by determining plasma endotoxin, zonulin, and antiendotoxin immunoglobulin IgG and IgM concentrations. Endotoxin is the major component of the outer membrane of gram-negative bacteria. Breakdown of the intestinal mucosal barrier resulted in the passage of endotoxin into the systemic circulation.^27^ In our study, the plasma endotoxin level was markedly decreased after using enteral glutamine for 10 days, compared with the control group. Our result is in line with studies of Peng et al.^12^ and Zhou et al.,^13^ which were conducted in severe burned patients after using enteral glutamine. According to the present study, glutamine supplementation was able to reduce plasma zonulin level. Zonulin as a master regulator of intercellular tight gap junctions is the only measurable physiological blood protein that reflects the intestinal permeability. Moreover, the increased zonulin levels are considered to be a marker of the impairment of intestinal barrier function.^28^ A recent study has shown that critically ill patients have elevated serum levels of zonulin-1.^29^ Klaus et al. demonstrated the elevated levels of plasma zonulin in septic patients.^30^ To our knowledge, zonulin is not considered as an outcome in previous clinical trials in critical care. Our results suggest that the zonulin assay is a reasonable choice to use as a marker for intestinal permeability in ICU-hospitalized patients. In this regard, L/M-ratio test provides challenging results according to the time of urine collection and fasting time, especially when considering that the participants in our study were the critically ill patients. Glutamine can promote plasma antiendotoxin IgG and IgM by effectively improving the immune system of patients. Consistent with our results, Wang et al. showed that after glutamine-enriched nutritional support in patients with advanced gastric cancer during perioperative chemotherapy, plasma IgG and IgM levels were significantly higher in the study group compared to the control group.^31^

In our study, there was also no discernible difference in the secondary outcomes relating to ICU-acquired sepsis, mortality, and LOS in ICU. These results support previous meta-analysis studies^32–34^ including the REDOX^35^ and METAPLUS^36^ trials indicating that glutamine supplementation given to a mixed population of critically ill patients does not significantly affect ICU mortality and LOS in ICU. In contrast, early systematic reviews have shown that glutamine supplementation may be associated with a reduction in complication and mortality rates and LOS in ICU in trauma, burn, and mixed ICU patients.^37,38^ Previous clinical studies have demonstrated that low plasma glutamine values (<420 µmol/L) upon admission are related to increased mortality.^39^ In our study, mean baseline glutamine was 488μmol/L and 27% of patients had glutamine values smaller than 420 µmol/L.

The majority of critically ill patients have at least one gastrointestinal symptom during their ICU stay.^40^ The reduced incidence of mucositis, diarrhea, and neuropathy with glutamine supplementation among patients under chemotherapy has been evidenced in recent studies.^41^ Our results showed that enteral glutamine supplementation decreases the occurrences of diarrhea, which is consistent with the result of a prospective randomized trial of enteral glutamine in critical illness.^42^ It suggests that the beneficial impact of glutamine supplementation on diarrhea may contribute to gastrointestinal mucosa rather than the host immune response.^43^ Our results showed that dietary enteral glutamine supplementation may decrease the occurrences of constipation. The mechanism of glutamine effect on constipation is thought to involve regulated endogenous gut microbiota, based upon an animal study.^44^

The most important strength of the current study was its design as a randomized, double-blind, placebo-controlled trial. The study also has some limitations. Body weight was recorded based on recent patient file information of subject's or relative's recall. The group of critically ill patients is very diverse, which makes it difficult to select a homogenous population with minimal variation in outcome parameters. The duration of ICU stay of patients also varies widely because some patients were tube-fed for only few days and others for more than a month. Another limitation is that the study patients were not followed up further to assess the sustainability of the results obtained after the termination of intervention.

## CONCLUSION

In conclusion, this study found some evidence that a glutamine-supplemented enteral diet in critically ill patients can decrease intestinal permeability and gastrointestinal complications. Despite this clear benefit, we found no effect on mortality at ICU and duration of stay in ICU. These findings should be confirmed in future randomized, placebo-controlled studies, which also include a washout period and larger sample sizes to analyze the subtypes of admission category in critically ill patients with glutamine deficiency.
